# Aging Across the Lifespan: Rethinking the David Barker Hypothesis

**DOI:** 10.1093/geroni/igab046.118

**Published:** 2021-12-17

**Authors:** Luigi Ferrucci

**Affiliations:** National Institute on Aging, Baltimore, Maryland, United States

## Abstract

For many years, the scope of geriatric medicine has been the care of older persons affected by multiple disease with the aim of improving their functional status and optimize quality of life. As our knowledge of the mechanisms of aging grows rapidly, it is becoming clear that accomplishing this scope requires taking a life-course perspective. Research have failed to establish a clear-cut distinction between normal aging and pathology leading to the hypothesis that aging is at the root of chronic diseases, and difference in health can be ascribed to different aging rates. Research in model organisms, suggest that aging can be modified with consequent changes on healthspan and longevity. Interventions that modulate aging may ultimately prevent most-age-associated diseases and their consequences. From this perspective, geriatric medicine is the leading and most promising branch of biomedical science. Challenges remain: first, demonstrating that certain interventions slow down the aging rate requires the valid measure of aging, and while many tools were developed, “epigenetic clocks” being the most promising, the underline mechanism that drive their changes with aging and validity in clinical applications are unclear. We do not know whether variability in the rate of biological aging assessed in old age are already detectable in younger individuals and person-specific rates remain constant during life. In 1986, David Barker stated the hypothesis that the period of gestation, characterized by a strong epigenetic imprinting, affects health and wellbeing across life, perhaps by setting the aging rate. Perhaps pediatric and geriatric medicine are more connected than previously believed.

